# Phenotypic and genomic characterization of *Bathyarchaeum tardum* gen. nov., sp. nov., a cultivated representative of the archaeal class *Bathyarchaeia*

**DOI:** 10.3389/fmicb.2023.1214631

**Published:** 2023-08-22

**Authors:** Maria A. Khomyakova, Alexander Y. Merkel, Dana D. Mamiy, Alexandra A. Klyukina, Alexander I. Slobodkin

**Affiliations:** ^1^Winogradsky Institute of Microbiology, FRC Biotechnology, Russian Academy of Sciences, Moscow, Russia; ^2^Faculty of Biology, Lomonosov Moscow State University, Moscow, Russia

**Keywords:** uncultured microorganisms, archaea, anaerobic, methoxylated aromatic compounds, *O*-demethylase, *Methanocalculus*

## Abstract

*Bathyarchaeia* are widespread in various anoxic ecosystems and are considered one of the most abundant microbial groups on the earth. There are only a few reports of laboratory cultivation of *Bathyarchaeia*, and none of the representatives of this class has been isolated in pure culture. Here, we report a sustainable cultivation of the *Bathyarchaeia* archaeon (strain M17C^Ts^) enriched from anaerobic sediment of a coastal lake. The cells of strain M17C^Ts^ were small non-motile cocci, 0.4–0.7 μm in diameter. The cytoplasmic membrane was surrounded by an S-layer and covered with an outermost electron-dense sheath. Strain M17C^Ts^ is strictly anaerobic mesophile. It grows at 10–45°C (optimum 37°C), at pH 6.0–10.0 (optimum 8.0), and at NaCl concentrations of 0–60 g l^−1^ (optimum 20 g l^−1^). Growth occurred in the presence of methoxylated aromatic compounds (3,4-dimethoxybenzoate and vanillate) together with complex proteinaceous substrates. Dimethyl sulfoxide and nitrate stimulated growth. The phylogenomic analysis placed strain M17C^Ts^ to BIN-L-1 genus-level lineage from the BA1 family-level lineage and B26-1 order-level lineage (former subgroup-8) within the class *Bathyarchaeia*. The complete genome of strain M17C^Ts^ had a size of 2.15 Mb with a DNA G + C content of 38.1%. Based on phylogenomic position and phenotypic and genomic properties, we propose to assign strain M17C^Ts^ to a new species of a novel genus *Bathyarchaeum tardum* gen. nov., sp. nov. within the class *Bathyarchaeia*. This is the first sustainably cultivated representative of *Bathyarchaeia*. We propose under SeqCode the complete genome sequence of strain M17C^Ts^ (CP122380) as a nomenclatural type of *Bathyarchaeum tardum*, which should be considered as a type for the genus *Bathyarchaeum*, which is proposed as a type for the family *Bathyarchaeaceae*, order *Bathyarchaeales*, and of the class *Bathyarchaeia*.

## Introduction

*Bathyarchaeia* is a cosmopolitan group of *Archaea* inhabiting diverse environments (Xiang et al., 2017; Zhou et al., 2018). Most often *Bathyarchaeia* are found in freshwater and marine sediments, where they could constituent a large portion of microbial communities. They have also been detected in soils, subsurface petroleum reservoirs, animal-associated habitats, and extreme environments such as terrestrial hot springs and deep-sea hydrothermal vents (Kubo et al., 2012; Fillol et al., 2016; Zhou et al., 2019). In many sediments, *Bathyarchaeia* are numerically dominant among *Archaea*, and it was argued that they are the most abundant *Archaea* on the planet (~2.0–3.9 × 10^28^ cells) (Kubo et al., 2012; Lloyd et al., 2013; He et al., 2016). The wide distribution and high abundance suggest that *Bathyarchaeia* may play an important role in the global biogeochemical cycles (Harris et al., 2018; Zhou et al., 2018). *Bathyarchaeia* are capable of degrading diverse organic compounds such as detrital proteins, plant-derived carbohydrates, lignin, and aromatic compounds (Meng et al., 2014; Lazar et al., 2016; Yu et al., 2018; Zhou et al., 2018; Yin et al., 2022). Moreover, members of this archaeal group have genomic markers for acetogenesis and methane metabolism, which are the most fundamental and ancient microbial biochemical energy-conservation processes (Evans et al., 2015; He et al., 2016; Loh et al., 2021). Several studies found genomic evidence that *Bathyarchaeia* could be involved in the nitrogen and sulfur cycles (Evans et al., 2015; Lazar et al., 2016; Zhang et al., 2016; Harris et al., 2018; Zhou et al., 2018). It was noticed that the distribution of different groups of *Bathyarchaeia* is related to geochemical parameters such as concentrations of total organic carbon, ammonium, and nitric oxide as well as to salinity and pH (Pan et al., 2019; Zou et al., 2020).

Current genome-based taxonomy considers *Bathyarchaeia* as a class, consisting of seven orders, within the phylum *Thermoproteota* (GTDB, Rinke et al., 2021). More recently, re-classification of *Bathyarchaeia* into eight order-level units was proposed (Hou et al., 2023). However, for a long time, *Bathyarchaeia* were classified based on 16S rRNA gene phylogeny into 25 subgroups, which demonstrated versatile physiological capabilities and possessed distinct ecological niches (Kubo et al., 2012; Fillol et al., 2016; Zhou et al., 2018). No pure cultures of *Bathyarchaeia* have been obtained so far, and the conclusions on their physiology and possible ecological roles are based on metagenomic data. Attempts to cultivate *Bathyarchaeia* are described only in a few studies. The enrichment cultures from freshwater estuarine sediment with a variety of carbon substrates were set up, and *Bathyarchaeia* affiliated with subgroup-4 and subgroup-8 were subcultured in these enrichments up to six transfers (Gagen et al., 2013). In another study, a 10-time increase in the gene copy numbers of *Bathyarchaeia* was demonstrated in incubations with lignin compared to the original sample. It was concluded that *Bathyarchaeia* of subgroup-8 were capable of assimilating CO_2_ autotrophically while utilizing lignin as an energy source (Yu et al., 2018). The combination of cell extraction, size fractionation, and roll-bottle technique resulted in the enrichment of *Bathyarchaeia* and two bacterial species on lignin (Hu et al., 2021). Stimulation of *Bathyarchaeia* in the initial enrichments by syringaldehyde, 4-hydroxybenzaldehyde, and vanillin under anaerobic conditions has been recently shown (Lin et al., 2022).

In this study, we obtained a highly purified culture of the representative of *Bathyarchaeia* (strain M17C^Ts^). The culture has high cell density and is amendable to regular subsequent transfers. Based on the results of cultivation experiments and metagenomic analysis, we determined the main cellular, physiological, and genomic characteristics of strain M17C^Ts^. This is the first study describing the cellular morphology and cell wall structure of the defined representative of *Bathyarchaeia;* also for the first time, a circular genome of *Bathyarchaeia* becomes available. To sustain the stability of nomenclature currently used for *Bathyarchaeia*, we formally describe strain M17C^Ts^ as *Bathyarchaeum tardum* gen. nov., sp. nov. under the SeqCode.

## Materials and methods

### Sampling site and cultivation conditions

Enrichment culture was obtained from a mixed sample of sediment and water collected in May 2017 from Lake Golubitskoe, located 150 m off the coast of the Azov Sea, Taman Peninsula, Russia. Temperature and pH values at the sampling site (45°19′47.7“N 37°15′48.7”E) were 20°C and 8.0–8.5, respectively, and NaCl concentration was 11 g l^−1^. Samples were taken anaerobically in tightly stoppered bottles and transported to the laboratory. An enrichment culture was initiated by inoculation of 10% (w/v) of the sample into anaerobically prepared (gas phase - N_2_, 100%), sterile (135°C, 1h), liquid medium of the following composition (per liter distilled water): KH_2_PO_4_, NH_4_Cl, KCl, MgCl_2_·6H_2_O (0.33 g each); CaCl_2_·6H_2_O, 0.033 g; NaCl, 20.00 g; NaHCO_3_, 2.00 g; 1 ml trace element solution (Slobodkin et al., 2012), 1 ml vitamin solution (Wolin et al., 1963), and resazurin solution (0.001g l^−1^). NaHCO_3_, vitamins, and Na_2_S·9H_2_0 (0.5 g l^−1^) were added after boiling and cooling the medium. The pH of the autoclaved medium was 8.0–8.5 at 20°C. 3,4-dimethoxybenzoic acid (DMB, 10 mM) was added as the substrate from anaerobically prepared sterile stock solution before inoculation. Ampicillin (1 g l^−1^) was used for the suppression of bacterial growth. The incubation temperature was 30°C.

### Phenotypic characterization of the isolate

Growth of the enrichment cultures was monitored by direct cell counting using a phase-contrast microscope (Olympus CX-43) followed by high-throughput sequencing of 16S rRNA gene amplicons for the prospective samples. Transmission electron microscopy was performed to determine cell morphology using JEM-100 electron microscope (JEOL, Japan).

All the cultivation experiments were performed in triplicate using Hungate tubes. Temperature (from 10 to 60°C) and pH growth ranges were determined using the same medium as mentioned before, with 10 mM DMB as a substrate and additives necessary for growth, inter alia yeast extract (0.1 g l^−1^), antibiotic mixture of streptomycin and lincomycin (0.05 g l^−1^ each), and sterilized (121°C, 30 min) sample of the natural sediment 0.2% (v/v). pH of the medium was adjusted to different values in the range of 5.0–10.0 with sterile anaerobic solutions of HCl (2 M) or NaOH (5%, w/v). To determine the NaCl requirement, varying amounts of NaCl (0–10.0%, w/v) were added directly into the Hungate tubes from pre-sterilized anaerobic stock solutions. Microaerobic growth was tested using the medium lacking the reducing agent, under 2% O_2_ (in N_2_) in the gas phase.

Electron donors and acceptors were added from sterile anaerobic stock solutions before inoculation. All the organic substrates (peptides, carbohydrates, organic acids, and methoxylated aromatic compounds) were filter-sterilized using 0.2 μm pore size syringe filters (Millipore). Growth with molecular hydrogen (10% in the gas phase) and carbon monoxide as the electron donor was tested in the presence of carbon dioxide as an electron acceptor. Molecular hydrogen, dimethyl sulfide, and methane formation were monitored by gas chromatography on a HayeSep N 80/100 mesh column at 40°C with argon as the carrier gas at 20 ml min^−1^ flow rate. Determination of nitrate and nitrite concentrations was held on a Stayer ion chromatograph (Aquilon) equipped with an IonPac AS22 column (Dionex, USA) and conductivity detector. Concentrations of DMB and 3,4-dihydroxybenzoate (DHB) were determined by ultra-performance liquid chromatography (UPLC)–UV analysis. The measurements were carried out on the equipment of the Shared-Access Equipment Center “Industrial Biotechnology” of the Federal Research Center “Fundamentals of Biotechnology” Russian Academy of Sciences. 0.1 ml of the sample was diluted with 0.9 ml of deionized water and used for UPLC analysis. UPLC-MS analysis was performed on Azura UVD 2.1S single wavelength UV/VIS detector (Knauer, Germany) equipped with A4061XB 10 μl analytical flow cell (Knauer, Germany) coupled to Elute UPLC (Bruker Daltonik, Germany) on Triart PFP 3 μm 2.1 × 150 mm 120 Å reverse phase column (YMC, Japan) with following conditions: gradient elution at 0.25 ml min^−1^ from 5% to 95% B in 10 min (A: 0.1% formic acid in water, B: 0.1% formic acid in acetonitrile), column at 30°C, 5 μl injection volume, and detection at 254 nm at 1 Hz rate. Data processing was performed in Compass DataAnalysis 5.1 (Bruker Daltonik, Germany).

### DNA extraction, 16S rRNA gene amplicon, and metagenome library preparation sequencing and analysis

DNA from mud samples and enrichment cultures was isolated using FastDNA Spin Kit for Soil according to the manufacturer's protocol (MP Biomedicals, Santa Ana, California, USA). The V4 region of the 16S rRNA amplicon libraries preparation, sequencing, and analysis was performed as previously described (Khomyakova et al., 2022). A shotgun metagenome library preparation and sequencing were done in BioSpark Ltd., Moscow, Russia, using KAPA HyperPlus Library Preparation Kit (KAPA Biosystems, UK), according to the manufacturer's protocol and NovaSeq 6000 system (Illumina, San Diego, CA, USA) with the reagent kit, which can read 100 nucleotides from each end. Raw reads were processed with Cutadapt (Martin, 2011) and Trimmomatic (Bolger et al., 2014) for adapter removal and quality filtering. Reads were processed in MetaWRAP (Uritskiy et al., 2018) using MetaSPAdes (Nurk et al., 2017) and MEGAHIT (Li et al., 2015) for assembly, MaxBin 2 (Wu et al., 2016), MetaBAT 2 (Kang et al., 2019), and CONCOCT (Alneberg et al., 2014) for binning and Salmon (Patro et al., 2017) for coverage calculation. Bin completeness and contamination were evaluated using CheckM (Parks et al., 2015). Taxonomies were assigned to each bin using GTDB-Tk (Parks et al., 2018). In the case when a more accurate phylogenetic analysis was needed, *de novo* phylogenetic trees were built using the list of 122 archaeal marker genes which were taken from GTDB (Parks et al., 2018). The trees were built using the IQ-TREE 2 program (Minh et al., 2020) with fast model selection via ModelFinder (Kalyaanamoorthy et al., 2017) and ultrafast bootstrap approximation (Minh et al., 2013) as well as approximate likelihood-ratio test for branches (Anisimova and Gascuel, 2006). All the sequencing data are deposited in NCBI BioProject PRJNA864620. Gene search and annotation were performed by BLAST (Altschul et al., 1990) and KEGG (Kanehisa et al., 2023) services. The MEROPS database (Rawlings et al., 2018) was used to identify putative proteases/peptidases in MAG M17C. The sequences identified as peptidases were collected and used as input for SignalP 6.0 (Teufel et al., 2022), to identify export signals. HydDB (Søndergaard et al., 2016) was used to classify the catalytic subunits of the hydrogenases.

### Quantitative polymerase chain reaction

For the quantification of *Bathyarchaeia*, a primer system for qPCR was designed using Primer-BLAST (Ye et al., 2012). The sequences of representatives of *Bathyarchaeia* that were used as targets for primers designed were obtained from our results of 16S rRNA gene-based profiling and metagenome sequencing. Sequences of the resulting primers were Bath2022F 5′- GGTAGGGGTGAAATCCTATAATCCCG−3′ and Bath2022R 5′- CCTCACCGTCGRGCGCGTTCTAG−3′. Product size−78 bp. According to TestPrime 1.0 (Klindworth et al., 2013), this primer pair is highly specific to a group of sequences from *Bathyarchaeia* class. The specificity of the primer system was also verified by sequencing the PCR product. For the calibration curve, linearized plasmid pAL2-T (Evrogen, Russia) with cloned M17C-01 16S rRNA gene fragment was used. qPCR analyses were carried out on a StepOnePlus Real-Time PCR System (Thermo Fisher Scientific, Waltham, MA, USA) and qPCRmix-HS SYBR (Evrogen, Russia). The amplification program consisted of the following steps: primary denaturation at 95°C for 5 min, followed by 30 cycles of denaturation at 95°C for 15 s, annealing at 64°C for 15 s, and elongation at 72°C for 12 s. The concentration of the primers was 0.25 μM each. Standards, samples, and negative controls were run in triplicate.

### Fluorescence *in situ* hybridization

Cells growing in the logarithmic phase were harvested by centrifugation and resuspended in 0.5 ml of phosphate-buffered saline (PBS) containing, in grams per liter, NaCl, 8.0; KCl, 0.2; Na_2_HPO_4_, 1.44; and NaH_2_PO_4_, 0.2 (pH 7.0). The cell suspension was mixed with 1.5 ml of 4% (w/v) freshly prepared paraformaldehyde solution (Sigma, Deisenhofen, Germany) and fixed for 1 h at room temperature. The cells were then collected by centrifugation (6,600 g for 1 min) and washed twice with PBS to ensure the removal of paraformaldehyde. The resulting pellet was resuspended in 0.5 ml of 50% ethanol–PBS (v/v). The *Bathyarchaeia*-specific oligonucleotide probe MCG493 (Kubo et al., 2012; 5′- CTTGCCCTCTCCTTATTCC-3′) was purchased from Syntol (Moscow, Russia). Hybridization was done on gelatin-coated (0.1%, w/v) and dried Teflon-laminated slides. The fixed cell sample was applied to these wells, hybridized to the fluorescent probe, and stained with the universal DNA stain 4',6-diamidino-2-phenylindole (DAPI, 1 mM) as described earlier (Dedysh et al., 2001). The specimens were examined with a ZeissAxioplan2 microscope (Zeiss, Jena, Germany) equipped with the Zeiss Filters No20 and 02 for Cy3-labeled probe and DAPI staining, respectively.

## Results

### Enrichment and cultivation of strain M17C^*Ts*^

**Figure 1:**
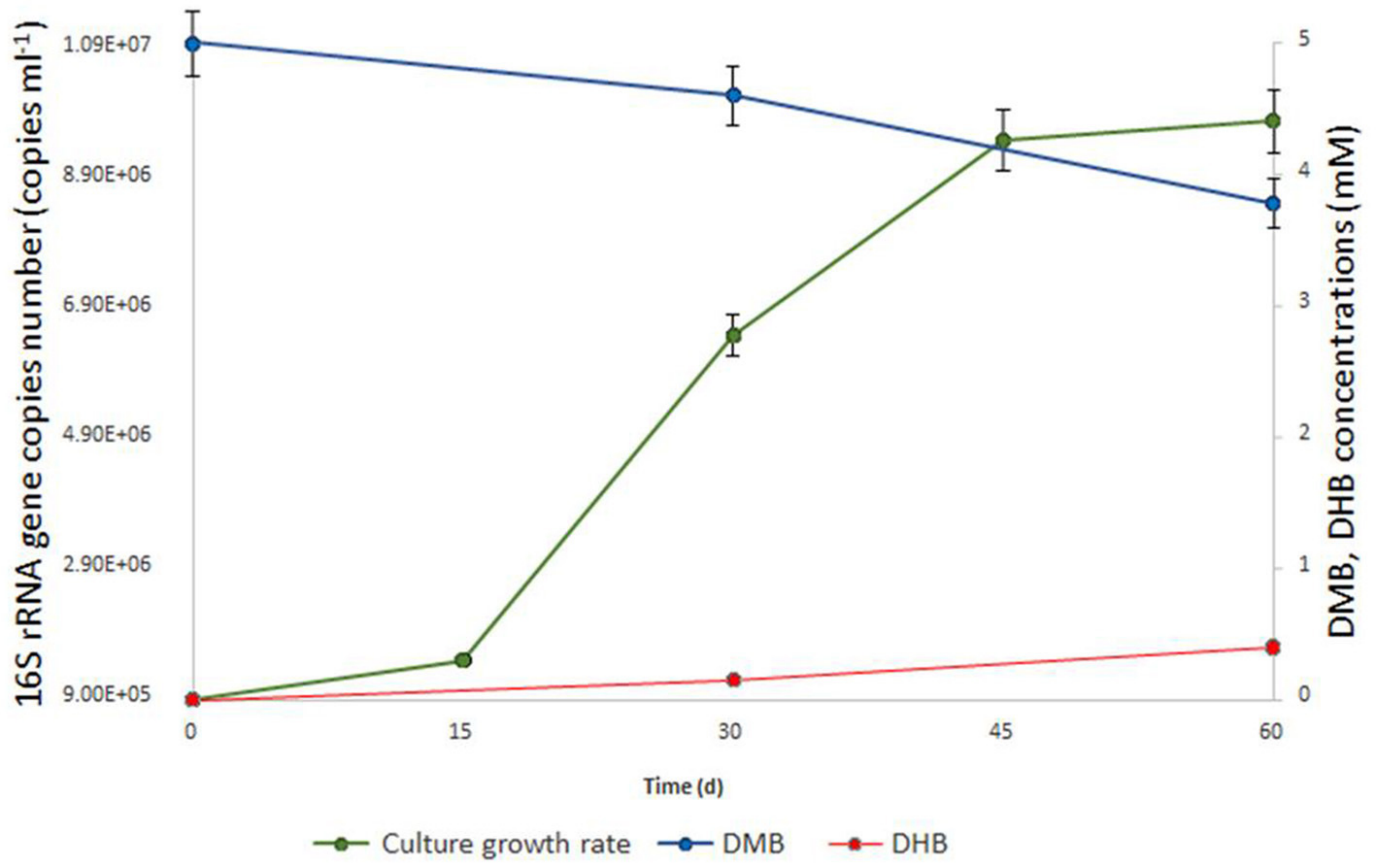
Growth dynamics of M17C^Ts^ cultivated with DMB (5mM). Culture was also supplemented with 0.1 g l^−1^ yeast extract, 0.20 ml 10 ml ^−1^ (v/v) of sterilized sediment from a natural sample and antibiotics mixture of streptomycin and lincomycin (0.05 g l^−1^ each). 1.2 mM of DMB (blue line) was consumed, and 0.4 mM of DHB (red line) was produced (in addition to acetate and ethanol) during 2 months of cultivation. No DHB, acetate, or ethanol was detected in un-inoculated controls after 2 months of incubation. The doubling time was estimated to be 7.2 days.

Enrichment cultures from anaerobic sediment of the lake Golubitskoe were set up by adding three different methoxylated aromatic compounds–2-methoxybenzoate, 2-methoxyphenol, and DMB. To suppress the growth of bacteria, ampicillin was added to the cultivation medium. Microbial growth was monitored by light-microscopic examinations followed by high-throughput sequencing of 16S rRNA gene amplicons as well as by parallel qPCR detection of *Bathyarchaeia* with specific primers. After 2 months of incubation, only the enrichment with DMB showed visible optical turbidity and growth of cells, mostly small non-motile cocci. The relative abundance of *Bathyarchaeia* in this enrichment was 43%. Attempts to obtain separate colonies were unsuccessful either with 1% Gelrite gellan gum or with 1.5% agar as the solidifying agents. We tried to purify *Bathyarchaeia* by serial dilution-to-extinction technique on a liquid medium. Overall, ten consequent 10% (v/v) transfers were made since the initial enrichment without loss of *Bathyarchaeia*. A detailed diagram of the transfers is shown in Supplementary Figure 1. Since the third transfer, we consider the bathyarchaeal component present in the cultures as a single population and designate it as strain M17C^Ts^. Finally, we obtain two highly purified cultures of strain M17C^Ts^ growing with DMB and casamino acids in the presence of antibiotics (mixture of lincomycin and streptomycin) and sterilized natural sediment (Supplementary material 1). The first culture contained only two archaeal species—strain M17C^Ts^ and *Methanocalculus alkaliphilus* (96% and 3% of relative abundance, respectively) (16S rRNA gene profiling data; BioSample SAMN34140869). This culture had an extremely slow growth rate (up to 6 months to reach the maximal cell density) and a low cell yield (1–2 x 10^6^ cells ml^−1^). The second culture grew faster (within 2–3 months) but contained more archaeal species. In addition to strain M17C^Ts^ (97.87%) and *Methanocalculus* (0.16%), the species of *Methanolobus* (0.38%), uncultured *Thermoplasmatota* (0.88% in total), and *Methanomicrobiaceae* (0.71%) were detected (shotgun metagenomic data; BioSample SAMN33973097). The doubling time for this second culture was estimated to be 7.2 days (Figure 1). Variations in substrate combinations and concentrations could probably improve growth rate or cell yield. At least the rates of growth were strongly dependent on the age and viability of the inoculum and could also vary depending on the composition of the microbial community, which was obvious for our cultivation lines.

### Cell morphology

**Figure 2:**
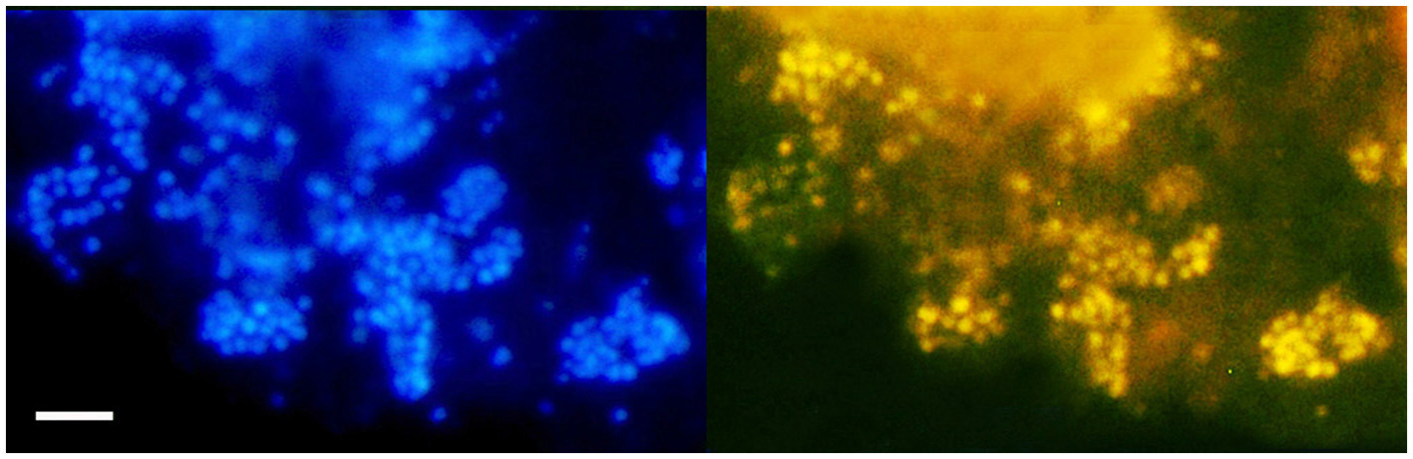
Fluorescence *in situ* hybridization (FISH) image of strain M17C^Ts^ cultivated with DMB. For FISH, M17C^Ts^ cells were hybridized with a *Bathyarchaeia* 16S rRNA targeted probe MCG493 (yellow fluorescence, right panel). After hybridization, the cells were counterstained with DAPI (blue fluorescence, left panel). Bar 1 μm.

**Figure 3:**
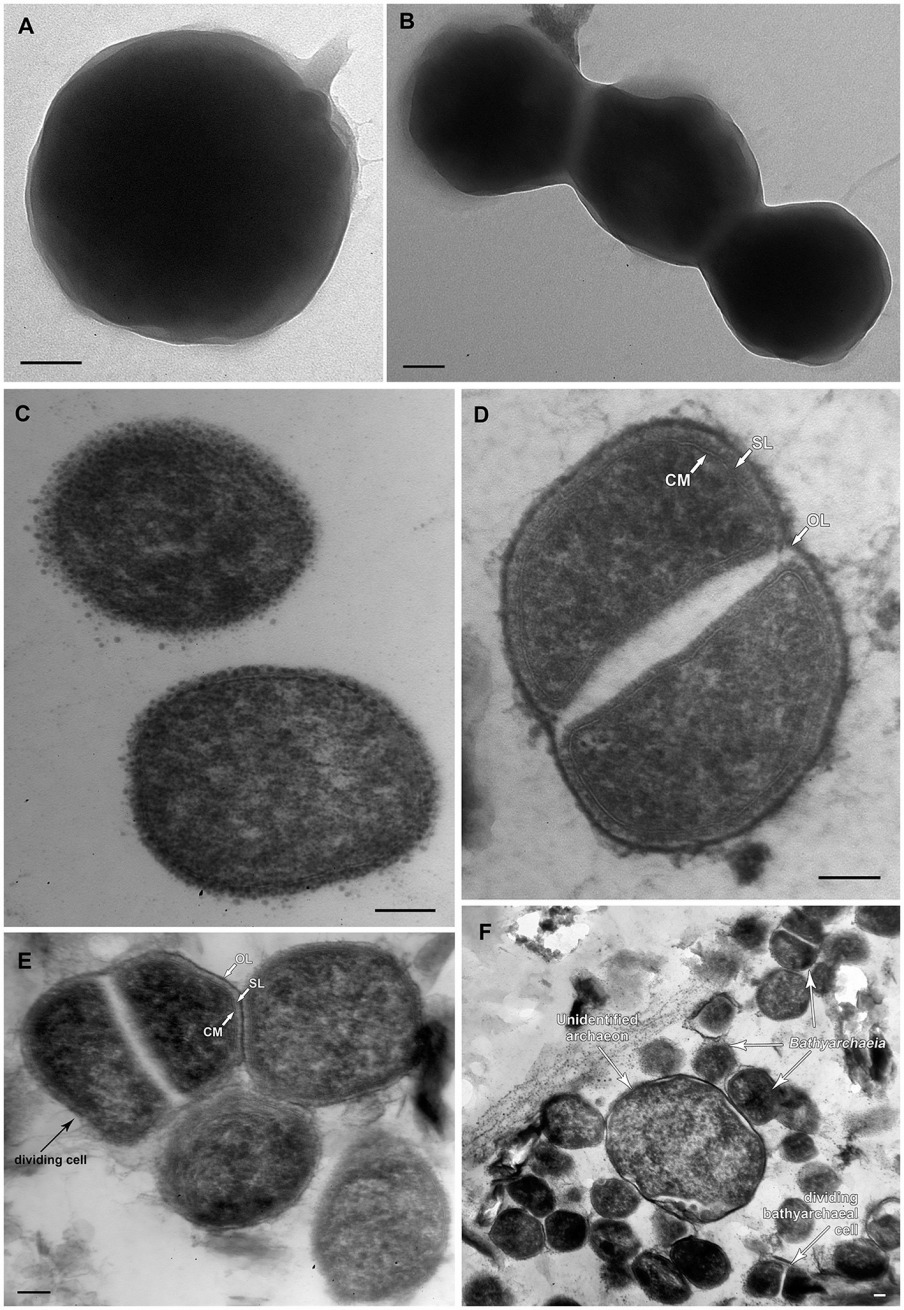
Morphological characteristics of strain M17C^Ts^. Electron micrographs of negatively stained cells showing **(A)** single cell of coccoid morphology with small areas of mucus on the surface of the cell; **(B)** chain of cells without surface appendages covered by unidentified common layer. Ultrathin section of **(C)** typical coccoid cells of strain M17C^Ts^ of regular shape; **(D, E)** dividing cells, **(F)** multiple cells of strain M17C^Ts^ and the single cell of the unidentified archaeon present in culture. CM—cytoplasmic membrane; SL—S-layer; OL—outermost surface layer. Bar 0.1 μm.

FISH analysis employing *Bathyarchaeia*-specific oligonucleotide probe MCG493 yielded a strong hybridization signal and demonstrated that the culture was predominantly represented by *Bathyarchaeia* (Figure 2). Transmission electron microscopy revealed that the cells of strain M17C^Ts^ were regular-shaped cocci with a diameter of 0.4–0.7 μm (Figure 3A). Flagella, pili, or other appendages on the surface of the cell were not observed, and motility was not detected. Chains of 3–5 cells covered by a common layer were occasionally formed (Figure 3B). The ultrathin section showed that the cells had an electron-dense layer, superimposing the cytoplasmic membrane, probably the S-layer typical for many *Archaea* (Figure 3C). In addition, the cells were covered by the outermost electron-dense layer, especially well-distinguishable during binary fission (Figures 3D, E). This outermost layer is probably a protein or polysaccharide sheath covering several individual cells. The cells of the strain M17C^Ts^ had a well-defined cytoplasmic membrane and no visible organelle-like structures. The cells of M17C^Ts^ were morphologically different from their co-culture partner *Methanocalculus alkaliphilus*, 0.4–0.7 μm in diameter regular cocci with binary division vs. 0.8–1.2 μm in diameter irregular cocci with division by septa, correspondingly (Figure 3F) (Zhilina et al., 2013; Sorokin et al., 2015).

### Physiological characteristics

Strain M17C^Ts^ was mesophilic and had an optimum growth temperature of 37°C and a temperature range for growth from 10°C to 45°C (Supplementary Figure 2A). The pH range for growth was 6.0–10.0, with an optimum at pH 8.0 (Supplementary Figure 2B). Growth of strain M17C^Ts^ was observed at NaCl concentrations of 0–6.0% (w/v) with an optimum at 2.0% NaCl (Supplementary Figure 2C).

Initial enrichments of strain M17C^Ts^ were performed in the medium containing DMB as a substrate, without the addition of other organic supplements. During the purification process, strain M17C^Ts^ gradually lost the ability to grow solely on DMB; however, the addition of 0.1 g l^−1^ yeast extract restored sustainable growth. Strain M17C^Ts^ was not subcultured if only yeast extract was added to the cultural medium.

To determine the compounds that can be used as growth substrates instead of DMB/yeast extract, we performed physiological experiments, in which the growth of strain M17C^Ts^ was monitored by real-time PCR along with high-throughput profiling of the 16S rRNA gene (Table 1).

**Table 1:** Effect of different compounds on growth of strain M17C^Ts^.

**Substrate**	**16S rRNA gene copies per ml of culture**	**Relative abundance of *Bathyarchaeia* in total community composition (%)**
**Support growth** ^a^		
Yeast extract (0.1 g l^−1^)	5.13E+06	90
Casamino acids (0.5 g l^−1^)	5.19E+06	90
Soytone (0.5 g l ^−1^)	6.01E+06	92
**Did not stimulate growth** ^a, b^		
Peptone (0.5 g l^−1^)	4.81E+05	59
Tryptone (0.5 g l^−1^)	2.97E+06	76
Glucose (0.5 g l^−1^)	1.79E+06	81
Fructose (0.5 g l^−1^)	3.31E+06	86
Sucrose (0.5 g l^−1^)	1.51E+06	85
Pyruvate (5mM)	8.12E+05	79
Lactate (5 mM)	4.23E+06	85
Chitin (0.5 g l^−1^)	9.49E+04	93
Casein (0.5 g l^−1^)	No growth	ND^*^
Cellulose (0.5 g l^−1^)	1.21E+02	ND^*^
Benzoate (5 mM)	4.05E+04	88
DHB (10 mM)	No growth	ND^*^
Formate (10 mM)	5.23E+03	2
Methanol (10 mM)	1.64E+05	4
Lignin (0.5 g l^−1^)	1.24E+06	94
2-methoxybenzoate (10 mM)	6.15E+03	78
2-methoxyphenol (10 mM)	1.76E+04	39
CO:CO_2_	1.91E+03	ND^*^
CO:CO_2_:H_2_	2.51E+03	9
CO_2_: H_2_	No growth	ND^*^
**Stimulate growth** ^a, b^		
Vanillate (10 mM)	1.58E+07	82
DMB (10 mM)	7.91E+06	84
**Potential electron acceptor** ^a, c^		
Sulfate (10 mM)	1.34E+05	64
Sulfite (5 mM)	3.35E+04	24
Thiosulfate (10 mM)	4.91E+03	63
DMSO (14 mM)	4.41E+07	91
S^0^ (5 g l^−1^)	No growth	ND^*^
Arsenate (10 mM)	1.19E+04	73
Nitrate (10 mM)	1.77E+07	90
Nitrite (2.5 mM)	7.10E+03	24
Fe (III) citrate (10 mM)	1.13E+03	17
AQDS (10 mM)	4.64E+04	73
Selenate (10 mM)	6.10E+06	84
Selenite (10 mM)	7.08E+03	21
Oxygen (2%, v/v)	No growth	ND^*^
**Antibiotics** ^a, c^		
Ampicillin (0.1 g l^−1^)	8.03E+05	81
Lincomycin (0.1 g l^−1^)	5.83E+05	77
Streptomycin (0.1 g l^−1^)	3.21E+06	87
Kanamycin (0.1 g l^−1^)	7.72E+05	67
2-bromoethane sulfonate (20 mM)	2.02E+03	33

a Incubation 60 days. All cultures were supplemented with 0.20 ml 10 ml ^−1^ (v/v) of sterilized sediment from a natural sample.

b In the presence of 0.1 g l^−1^ yeast extract, with the exception of peptone and tryptone variants.

c In the presence of 0.1 g l^−1^ yeast extract and DMB as an electron donor.

* ND—not determined.

No growth stimulation was observed when the concentration of yeast extract was increased up to 0.5 g l^−1^ compared to 0.1 g l^−1^. Yeast extract could be replaced by casamino acids or soytone but not by tryptone or peptone. Glucose, fructose, sucrose, lactate, pyruvate, lignin, chitin, casein, cellulose, formate, methanol, benzoate, DHB, 2-methoxyphenol, 2-methoxybenzoate, molecular hydrogen, and carbon monoxide did not stimulate growth (Table 1). From tested compounds, only DMB and vanillate stimulated the growth of strain M17C^Ts^. Products of growth on DMB and yeast extract were DHB, acetate, and ethanol. 1.2 mM of DMB was consumed, and 0.4 mM of DHB was produced during 2 months of cultivation. No DHB, acetate, or ethanol was detected in non-inoculated controls after 2 months of incubation. Small amounts of methane (<0.5 mM) were produced by methanogenic partners.

Among tested potential electron acceptors, dimethyl sulfoxide (DMSO) and nitrate stimulated the growth of M17C^Ts^. Sulfate, thiosulfate, antraquinone-2,6-disulfonate (AQDS), selenate, selenite, arsenate, Fe (III)-citrate, elemental sulfur, sulfite, nitrite, or oxygen (2% (v/v) in the gas phase did not stimulate growth. Oxygen and elemental sulfur had a profound inhibitory effect (Table 1).

Of the antibiotics tested, strain M17C^Ts^ showed the highest resistance to streptomycin (0.1 g l^−1^) (Table 1).

### Phylogenomic analysis and environmental distribution

**Figure 4:**
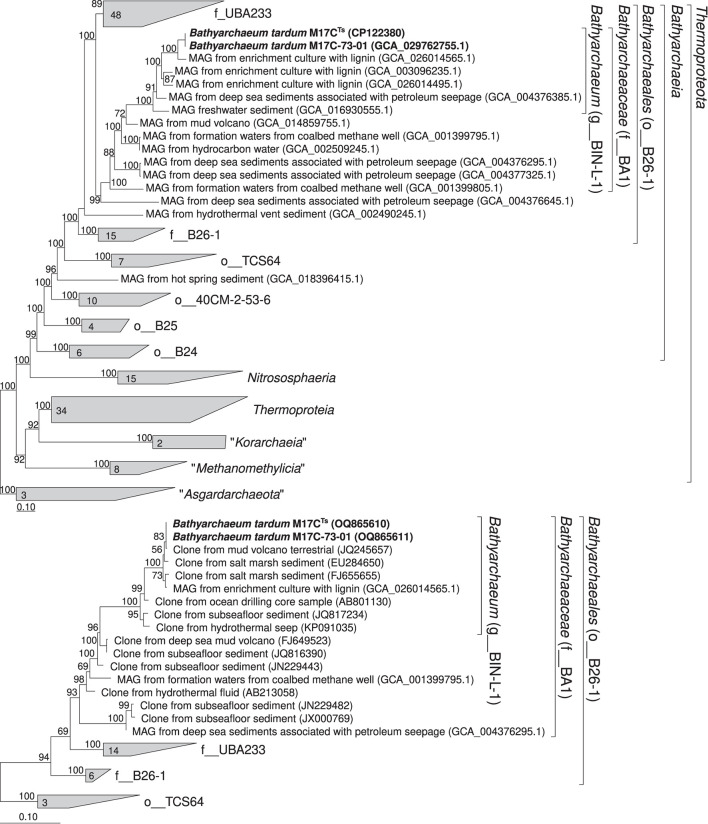
Phylogenomic placement of M17C-01^Ts^ and M17C-73-01 MAGs based on **(A)** concatenated partial amino acid sequences of 122 archaeal single copy conserved marker genes with taxonomic designations according to the GTDB (Parks et al., 2018); **(B)** on 16S rRNA gene sequences. The “g_” stands for genus level, “f_”—for family level, and “o_”—order level. The trees were built using the IQ-TREE 2 program (Minh et al., 2020) with fast model selection via ModelFinder (Kalyaanamoorthy et al., 2017) and ultrafast bootstrap approximation (Minh et al., 2013) as well as approximate likelihood-ratio test for branches (Anisimova and Gascuel, 2006). Bootstrap consensus tree is shown with values placed at the nodes. Bar, 0.1 changes per position.

According to our phylogenomic reconstruction based on 122 archaeal single copy conserved marker genes (Parks et al., 2018), M17C^Ts^ (M17C-01 MAG) is a part of BIN-L-1 genus-level lineage (Figure 4A) which belongs to the BA1 family-level lineage and B26-1 order-level lineage according to GTDB (Parks et al., 2018). The B26-1 group is formerly referred to as Bathy-8 (Yu et al., 2017). BIN-L-1 cluster contains several MAGs from enrichment cultures growing with lignin as well as MAG from sediments associated with petroleum seepage and freshwater sediments (Yu et al., 2018). These data indicate the possible specialization of representatives of this genus-level group to growth on natural methoxylated compounds. The phylogenetic position of M17C^Ts^ revealed by 16S rRNA gene-based phylogenetic reconstruction agrees well with the phylogenomic data (Figure 4B).

We analyzed the distribution of BIN-L-1 cluster members based on 16S rRNA gene sequence data available from public databases. For this purpose, we selected sequences in GenBank that have more than 94.5% similarity to the 16S rRNA gene sequences of MAGs of this group (Yarza et al., 2014). This selection consisted of 457 sequences. Most of the sites where these microorganisms were found belonged to the marine sediments often associated with the presence of terrigenous organic matter, e.g., terrigenous deposits, estuary (Li et al., 2012), and sunken woods (Fagervold et al., 2012). The second largest group of ecotopes where BIN-L-1-related sequences have been found are the sites associated with methane seepages, e.g., hydrate-bearing sediments (Dang et al., 2009), cold seep sediment (Li et al., 1999), and mud volcanoes (Cheng et al., 2012). Soils and freshwater sediments were less significant environments for BIN-L-1-related sequences.

### Metagenome statistics

For metagenomic analysis, two enrichment cultures from different cultivation stages, grown on DMB and antibiotics mixture with different proteinaceous additives, were taken (Supplementary Figure 1). The first metagenome (M17C) was analyzed after the third transfer on DMB and ampicillin, where the relative abundance of *Bathyarchaeia* group according to 16S rRNA gene-based profiling reached 74%, and the second metagenome (M17C-73) was taken from the eight transfer on DMB, antibiotics, and casamino acids (here *Bathyarchaeia* group built up 98%) since the richness by the group of interest as well as its growth rates was the highest of the achieved.

The main assembly quality indicators for M17C and M17C-73 metagenomes are shown in Supplementary Table 1. An overview of all MAGs is shown in Table 2. As a result of M17C metagenomic analysis, we were able to assemble the genome of the *Bathyarchaeia* (MAG M17C-01) to the level of a complete circular chromosome of the size of 2,152,572 bp (98.28% completeness and 0.934% contamination according to CheckM). The nomenclatural type is CP122380^Ts^ according to SeqCode requirements (Hedlund et al., 2022; Whitman et al., 2022). Its relative abundance in the metagenome was 83.38%. In addition to the M17C-01 *Bathyarchaeia*, the M17C metagenome also contained uncultured representatives of *Acholeplasmataceae* (isolated later as *Mariniplasma* sp.) and *Dethiobacteraceae* families (4.51% and 3.03%, respectively) as well as representatives of the *Methanothrix* and *Soehngenia* genera (3.29% and 3.61%, respectively).

**Table 2:** Overview of all MAGs >50% complete and with <5% contamination.

**Bin ID**	**Domain**	**Taxon**	**Abundance %**	**Completeness %**	**Contamination %**	**# contigs**	**Genome Size, bp**
**M17C**							
M17C-01	A	*Bathyarchaeia*	83.38	98.28	0.934	1	2152501
M17C-02	B	*Acholeplasmataceae*	4.51	98.66	0.444	41	1893757
M17C-03	B	*Dethiobacteraceae*	3.03	93.77	2.824	556	2658718
M17C-04	A	*Methanothrix*	3.29	98.68	0	241	2087961
M17C-05	B	*Soehngenia*	3.61	98.6	2.097	116	2549159
**M17C-73**							
M17C-73-01	A	*Bathyarchaeia*	97.87	97.82	0.934	12	2139529
M17C-73-02	A	*Methanomicrobiaceae*	0.71	99.54	0	28	2205140
M17C-73-03	A	*Methanolobus*	0.38	100	3.267	58	2429529
M17C-73-04	A	*Thermoplasmatota* (EX4484-6)	0.33	94	4	102	2963565
M17C-73-05	A	*Thermoplasmatota* (DHVEG-1)	0.24	87.33	3.2	161	1576231
M17C-73-06	A	*Thermoplasmatota* (DHVEG-1)	0.16	71.81	3.52	456	1839870
M17C-73-07	A	*Methanocalculus*	0.16	91.77	1.307	578	2306789
M17C-73-08	A	*Thermoplasmata*	0.15	89.88	2.042	518	1762735

The genome of the *Bathyarchaeia* from the M17C-73 metagenome (MAG M17C-73-01) was very close but not identical to M17C-01: ANI value is 99.31%; in addition, both MAGs contain identical 16S rRNA genes. Apparently, we see here a case of microdiversity in *Bathyarchaeia* population. The relative abundance of the M17C-73-01 MAG in the M17C-73 metagenome reached 97.87%. The rest of the community was represented only by archaea: by methanogens of the *Methanolobus* and *Methanocalculus* genera as well as of *Methanomicrobiaceae* family and by uncultivated representatives of the “*Candidatus* Thermoplasmatota” phylum including archaea of the DHVEG-1 and EX4484-6 phylogenetic clusters. They all represented only a minor (<1%) part of the community. M17C-01 genome was chosen as a type genome and further analyzed to explain the revealed physiological features of the M17C^Ts^ isolate.

### Metabolism of proteinaceous substrates

Our physiological experiments demonstrate the ability of strain M17C^Ts^ to metabolize proteinaceous substrates. Growth on amino acids typically requires the gluconeogenic pathway for carbohydrate synthesis (Danson et al., 2007) and in line with that all genes for a reverse glycolytic pathway, including pyruvate:ferredoxin oxidoreductase and fructose 1,6-bisphosphatase, have been identified in the genome. However, glucose-6-phosphatase is absent, which makes it impossible to synthesize glucose from phosphorylated form. The genome of strain M17C^Ts^ contained genes for the degradation of detrital proteins (i.e., multiple membrane ABC transporters, peptidases, aminotransferases, and ferredoxin oxidoreductases) as well as genes encoding intracellular archaeal proteasome complex (WGM88999; WGM89238; WGM90643) and several presumably extracellular peptidases of M24 and M42 families (WGM88659 and WGM89452 correspondingly). All these proteins are intracellular which is consistent with the data on the deficiency of extracellular proteases in *Bathyarchaeia* (Berben et al., 2022). Apparently, utilization of protein compounds by strain M17C^Ts^ is limited to amino acids, which are small enough for immediate transmembrane uptake. Numerous solute-binding protein subunits of ABC-type transporters were encoded in the genome of strain M17C^Ts^; their physiological substrates are difficult to predict based solely on the sequence information. The genome of strain M17C^Ts^ encoded several genes of aminotransferases, in particular, aromatic amino acid aminotransferase and histidinol-phosphate aminotransferase (WGM89691; WGM89080). Degradation of the amino acid backbone proceeds through donor:ferredoxin oxidoreductases, yielding reduced ferredoxin as the final product. In the genome of strain M17C^Ts^, we identified genes of aldehyde:ferredoxin oxidoreductase, 2-oxoacid:ferredoxin oxidoreductase, and pyruvate:ferredoxin oxidoreductase (Supplementary Table 2). Pyruvate produced in aminotransferase reaction can be further oxidized by pyruvate:ferredoxin oxidoreductase (WGM89686-89) to acetyl-CoA, which can be oxidized with ATP production by acetyl-CoA synthetase (WGM89748). Acetate kinase and phosphate acetyltransferase responsible for substrate-level phosphorylation in fermenting and acetogenic bacteria are absent from the genome strain M17C^Ts^. The pathways for the formation of ethanol, which was found along with acetate as a metabolic product of strain M17C^Ts^, remain unclear. Genes of alcohol dehydrogenase were absent from the genome (Supplementary Table 2); however, genes of aldehyde:ferredoxin oxidoreductase playing an important role in alcohol-producing anaerobes (Nissen and Basen, 2019) were present (WGM89453). Acetogenesis may fulfill the role of the redox sink, recycling reduced ferredoxin produced by amino acid degradation.

Inspite of the presence of the genes for a complete Embden–Meyerhof–Parnas (glycolysis) pathway (Supplementary Table 2), in physiological experiments, strain M17C^Ts^ did not show the ability to metabolize carbohydrates. The reason for this could be the absence of sugar transporters.

### Metabolism of aromatic compounds

The isolation process together with physiological experiments has demonstrated the ability of strain M17C^Ts^ to grow on methoxylated aromatic compounds such as DMB and vanillate. We conducted a manual BLASTP search for *O*-demethylase genes involved in methoxydotrophic growth and found a putative cluster of three neighboring genes (WGM89983-84; WGM89986), previously described in *Bacteria* and *Archaea* (Kurth et al., 2021; Welte et al., 2021). Activating enzyme (AE), required for the reduction of corrinoid protein after an inadvertent oxidation of the corrinoid cofactor, was not found in the genome. On the other hand, its presence is not a prerequisite for the *O*-demethylase reaction. For example, *Moorella thermoacetica* does not possess AE, and its *O*-demethylase is a three-component system; in this case, the reductive activation system is not required (Naidu and Ragsdale, 2001). As the methyltransferases and corrinoid protein are cytosolic, strain M17C^Ts^ needs transporters for the uptake of methoxylated aromatic compounds. Previous studies have characterized archaeal putative aromatic acid:H^+^ symporter belonging to the major facilitator superfamily from *Archaeoglobus fulgidus* grown on 2-methoxyphenol (Welte et al., 2021). We also found in the genome of strain M17C^Ts^ gene WGM90668, which demonstrates high amino acids similarity with one of MFS transporters from *Archaeoglobus fulgidus*, probably involved in the import of the methoxylated aromatic compounds and export of the hydroxylated derivatives. However, in strain M17C^Ts^ genome, this gene is located apart from a putative *O*-demethylase cluster.

Attempts to detect in the genome of strain M17C^Ts^ genes of benzoyl-CoA reductase (Bcr), a key enzyme of anaerobic benzoate catabolism, revealed the presence of a cluster of three adjacent genes, encoding BcrACD, while gene of BcrB, a part of substrate-activating module, was not detected (Supplementary Table 2). In our growth experiments, it was shown that benzoate and DHB inhibited the growth of strain M17C^Ts^ (Table 1). Genes of corresponding aromatic ligases and reductases were absent from the genome. Several putative transporters of aromatic compounds were found (WGM90668; WGM89538).

The methyl group from the methoxylated substrate most probably enters the Wood–Ljungdahl pathway (WLP), leading to acetogenesis, since a key enzyme of the methanogenic pathway (McrA) was not encoded in the genome. The strain M17C^Ts^ genome contained a complete set of WLP genes in the archaeal version, involving methanofuran and tetrahydromethanopterin (THMPT) as C_1_ methyl group carriers (Supplementary Table 2). The THMPT branch might function in either oxidation or reduction directions. In the oxidation direction, methylene is oxidized to CO_2_, producing ATP while reducing NAD^+^ and ferredoxin. Hox hydrogenase, found in the genome (WGM89841-43; WGM90709), can catalyze hydrogen production for cofactor regeneration and disposal of excess reductants, including those that would be produced by the THMPT branch working in the oxidation direction. In the reduction direction, the branch could serve for carbon fixation.

### Utilization of electron acceptors

During physiological experiments, we found that the addition of DMSO and nitrate stimulated the growth of strain M17C^Ts^. Dimethyl sulfide was formed as a product of DMSO reduction (up to 2.3 mM). The enzymes involved in DMSO and nitrate reduction in strain M17C^Ts^ remain unclear. Electron shuttling via a cysteine–cystine couple, characteristic for *Thermococcus* species, growing on peptides and DMSO (Choi et al., 2016), seems to be incomplete, and only WGM89435 gene encoding thioredoxin reductase was identified. Genes of DMSO reductase of acetogenic bacterium *M. thermoacetica*, composed of three subunits (Rosenbaum et al., 2022), were not found except the genes encoding putative subunit DmsB (WGM89217; WGM89773).

The consumption of nitrate was relatively low, and 0.5–0.7 mM of nitrate was consumed from the initial concentration of 10 mM. Nitrite and N_2_O were not formed. Genes coding for periplasmic nitrate reductase (NapAB) were not found in the genome of strain M17C^Ts^. The genes of catalytic and quinol-oxidizing subunits (NarGI) of another dissimilatory nitrate reductase, a membrane-bound Nar-type nitrate reduction complex, were also absent from the genome. One gene (WGM89217) demonstrated 33% amino acid sequence identity (52.7% similarity) to the electron-transfer subunit NarH from archaeon *Haloferax mediterranei*. A gene homologous to assimilatory nitrate-reductase NasA was found (WGM89749), which demonstrated 22.5% amino acid sequence identity (51.7% similarity) to NasA from *Haloferax medditerranii* (Martínez-Espinosa et al., 2001). No other Nas subunits were identified. The genome of strain M17C^Ts^ harbors several other nitrogen-related genes encoding ammonium transporter (*amt*, WGM90598) and hydroxylamine reductase (*hcp*, WGM90665).

The genome of strain M17C^Ts^ had a gene cluster with high homology to NADH:quinone oxidoreductases (Nuo); only NuoJ-encoding gene was not found in the genome. On the other hand, several genes from this cluster resemble catalytic subunits of [NiFe] hydrogenases of subgroup-4. It was proposed recently that in *Bathyarchaeia* of subgroup 6, this cluster is not a respiratory complex but is instead a novel energy-converting Hfo hydrogenase (Loh et al., 2021). Both coordination sites of the [NiFe] cofactor (L1 and L2 motifs; Vignais and Billoud, 2007), which are no longer conserved in NuoD, are present in WGM88738 (annotated as NuoD in GenBank, Supplementary Figure 3). Genes encoding the NuoEFG subunits known to form the domain involved in NADH binding and oxidation seem to be present in the genome of strain M17C^Ts^ and are organized in one cluster, suggesting the ability of this complex to accept electrons from NADH (WGM89840-42; Sazanov, 2007). The genome of strain M17C^Ts^ also has genes, encoding six of the eight subunits of the archaeal V-type ATP synthase, and a primitive respiration can be possible as described for another archaeon (Sapra et al., 2003).

## Discussion

Since the discovery of *Bathyarchaeia* two decades ago, versatile metabolic capabilities such as acetogenesis, methanogenesis, and fermentation have been suggested for its members; however, none of these physiological functions have been directly confirmed due to the lack of laboratory cultures (Inagaki et al., 2003; Evans et al., 2015; Zhou et al., 2018). We have obtained the first highly purified culture with a relative abundance of *Bathyarchaeia* 98%. To date, morphological characteristics of *Bathyarchaeia* were not investigated. We have demonstrated that the cells of the bathyarchaeal component of the culture (strain M17C^Ts^) are small non-motile cocci, and the chains of the cells are covered with an outer sheath of unknown composition. Combining physiological studies with genome analyses, we found that strain M17C^Ts^ is an anaerobic, slow-growing archaeon, capable of utilization of complex proteinaceous substrates and methoxylated aromatic compounds.

The ability of archaea to transform methoxylated aromatic compounds, which are important components of lignin, the second most abundant polymer on the earth, was unknown for a long time and was demonstrated only recently (Mayumi et al., 2016; Welte et al., 2021). The central carbon metabolism of strain M17C^Ts^ during methoxydotrophic growth most probably involves the oxidation of methyl groups of 3,4-dimethoxybenzoate or vanillate, reduction of CO_2_, and production of acetate. Utilization of DMB and vanillate as growth substrates is strongly supported by the presence of *O*-demethylase gene cluster in the genome. The confinement of the closest relatives of strain M17C^Ts^ to the sources rich in lignin, as well as to petroleum seepages, confirms the dependence of this group on aromatic compounds. Strain M17C^Ts^ does not use carbohydrates and is not capable of lithotrophic growth. Most probably, its ecological function in anaerobic sediments is the utilization of low concentrations of oligomers derived from plants and animals necromass.

According to metagenomic analyses, anaerobic respiration is not common in *Bathyarchaeia* and is only suggested for nitrogen compounds (Lazar et al., 2016; Harris et al., 2018; Deb and Das, 2022). In particular, genes encoding periplasmic nitrate reductase (*narH*) and nitrite reductase (*nrfHA*) were found in the genome of “*Candidatus* Bathyarchaeota” BE326-BA-RLH, suggesting the potential for dissimilatory nitrate reduction (Harris et al., 2018). We have observed ~2.5-time stimulation of growth of strain M17C^Ts^ by nitrate. However, genes encoding catalytic subunits of dissimilatory nitrate reductases were not found in the genome. The reason for growth stimulation by nitrate is not clear, and low amounts of nitrate were consumed which may indicate its assimilation or use as an electron acceptor in energy metabolism by yet unknown biochemical mechanisms.

Another potential electron acceptor that stimulates the growth of strain M17C^Ts^ is DMSO. This methylated sulfur compound plays a significant role in the biogeochemical cycle of dimethyl sulfide (Xiong et al., 2016). In *M. thermoacetica*, the reduction of DMSO may be associated with bacterial acetogenesis where DMSO can be used alongside CO_2_ as an electron acceptor (Rosenbaum et al., 2022). We did not find genetic determinants of this process (neither Dms-type nor Dor-type) in the genome of strain M17C^Ts^. No obvious genomic determinants of terminal reductases and of electron transport chain components such as cytochrome oxidases, quinones, or c-type cytochromes were detected, so the ability of strain M17C^Ts^ to conserve energy by anaerobic respiration is not evident.

Strain M17C^Ts^ probably has mutualistic relationships with methanogenic component of the cultures. In nature, *Bathyarchaeia* are often co-occurred with *Methanomicrobia*, where they are localized in close association (Collins et al., 2005; Xiang et al., 2017). Multiple deficiencies in purine and pyrimidine biosynthesis as well as in the synthesis of cofactors, including CoF_420_ (taking part in WLP) and vitamins such as cobalamin which is necessary for *O*-demethylase reaction, were found in the genome of strain M17C^Ts^ (Supplementary Table 2). Our experiments show that the addition of an inhibitor of methanogenesis 2-bromoethane sulfonate suppressed the growth of strain M17C^Ts^ (Table 1). Thus, strain M17C^Ts^ could be obligatory dependent on a methanogenic partner, *Methanocalculus alkaliphilus*, in acquiring the growth factors necessary for metabolism. Meanwhile, *Methanocalculus alkaliphilus* receives molecular hydrogen produced by strain M17C^Ts^ as a growth substrate.

This study expands our knowledge of the diversity, metabolic functions, and possible ecological role of *Bathyarchaeia*. Based on phylogenetic position, phenotypic, physiological, and genomic properties, we propose to assign strain M17C^Ts^ to the novel taxa on the species, genus, family, order, and class levels. To preserve the currently widely used and stable name for this group of archaea, we also propose that the term *Bathyarchaeia* (Meng et al., 2014) be retained for all new taxa.

### Description of *Bathyarchaeumtardum*^*Ts*^

*Bathyarchaeum tardum* (tar'dum. L. neut. adj. *tardum*, slow, referring to its slow growth).

Cells are small, non-motile cocci, with a diameter of 0.4–0.7 μm. Occasionally forms chains of 3–5 cells covered by a common sheath. The cytoplasmic membrane is surrounded by an S-layer. Strictly anaerobic. Mesophilic. Grows at 10–45°C (optimum 37°C), at pH 6.0–10.0 (optimum 8.0), and at NaCl concentrations of 0–60 g l^−1^ (optimum 20 g l^−1^). Grows on 3,4-dimethoxybenzoic acid, vanillate in the presence of yeast extract, casamino acids, or soytone. Does not utilize glucose, fructose, sucrose, lactate, pyruvate, lignin, chitin, peptone, tryptone, casein, cellulose, formate, methanol, benzoate, 3,4-dihydroxybenzoate, 2-methoxyphenol, 2-methoxybenzoate, molecular hydrogen, and carbon monoxide. Dimethyl sulfoxide and nitrate stimulate growth, while oxygen, elemental sulfur, carbon monoxide, and molecular hydrogen inhibit growth. Does not reduce sulfate, thiosulfate, antraquinone-2,6-disulfonate, selenate, selenite, arsenate, Fe(III)-citrate, elemental sulfur, sulfite, nitrite, or oxygen. Growth depends on addition of sterile natural sediment. The complete genome of strain M17C^Ts^, available under the GenBank assembly accession number (CP122380^Ts^), is the designated nomenclatural type for the species and was recovered from an enrichment culture, cultivated on 3,4-dimethoxybenzoic acid and established from the anaerobic sediment of a coastal lake at the Taman Peninsula, Russian Federation. The genome has the size of 2.15 Mb and a G + C content of 38.1%. Completeness is estimated by CheckM at 98.28% with 0.934% contamination. *Bathyarchaeum tardum* is also presented by high-quality MAG M17C-73-01 (GCA_029762755.1; Genome size-2139529bp, # of contigs–12, completeness–97.82%, contamination–0.934%). Both MAGs have identical full-length 16S rRNA genes, and ANI value between MAGs is 99.31%. With the other MAGs of the *Bathyarchaeum* genus, which were included in this genus based on our phylogenetic reconsideration (Figure 4), they have an ANI value no higher than 84.5%.

### Description of the genus *Bathyarchaeum*

Ba.thy.ar.chae'um (Gr. masc.adj. *bathys*, deep as it locates deep phylogenetic branching within *Archaea*; N.L. neut. n. *archaeum*, ancient one, archaeon; from Gr. masc. adj. *archaîos*, ancient; N.L. neut. n. *Bathyarchaeum*, deeply branched archaeon).

High-quality MAGs of this genus have been assembled from enrichment cultures with 3,4-dimethoxybenzoic acid inoculated from anaerobic sediment of a coastal lake at the Taman Peninsula, from enrichment cultures with lignin inoculated from coastal sediments of northern East China Sea, from deep-sea sediments associated with petroleum seepage (Atlantic Ocean), and from sediment of high-sulfide freshwater Zodletone spring (Oklahoma, USA). Based on 16S rRNA gene sequence data, microorganisms of this genus were often found in the marine sediments associated with the presence of terrigenous organic matter, e.g., terrigenous deposits, estuary (Li et al., 2012), and sunken woods (Fagervold et al., 2012) and in ecotopes associated with methane seepages, e.g., hydrate-bearing sediments (Dang et al., 2009), cold seep sediment (Li et al., 1999), and mud volcanoes (Cheng et al., 2012). AAI values among genomes representing separate species within the genus range between 71.87% and 83.83%. Our phylogenomic reconstruction (Figure 4) supports delineation of the genus *Bathyarchaeum*. The Relative Evolutionary Divergence (RED) in phylogenomic reconstruction of GTDB 207 for the genus *Bathyarchaeum* (g__BIN-L-1) is 0.931.

The nomenclatural type of the genus is *Bathyarchaeumtardum*^Ts^.

### Description of the family *Bathyarchaeaceae*

Ba.thy.ar.chae.a.ce'ae (N.L.fem.n. *Bathyarchaeum*, type genus of the family; L. fem. pl. suff. *-aceae*, ending to denote a family; N.L. fem. pl. n. *Bathyarchaeaceae*, family of the genus *Bathyarchaeum*).

Our phylogenomic reconstruction (Figure 4) supports delineation of the family *Bathyarchaeaceae*. RED in phylogenomic reconstruction of GTDB 207 for the family *Bathyarchaeaceae* (f__BA1) is 0.691. AAI values range between 59.6 and 70.8% among members of different genera of this family. The nomenclatural type of the family is the genus *Bathyarchaeum*.

### Description of the order *Bathyarchaeales*

Ba.thy.ar.chae.a'les (N.L. fem. n. *Bathyarchaeum*, type genus of the order; N.L. fem. pl. suff. *-ales*, ending denoting an order; N.L. fem. pl. n. *Bathyarchaeales*, order of the genus *Bathyarchaeum*, type genus of the order).

Our phylogenomic reconstruction (Figure 4) supports delineation of the order *Bathyarchaeales*. RED in phylogenomic reconstruction of GTDB 207 for the order *Bathyarchaeales* (o__B26-1) is 0.514. The nomenclatural type of the order is the genus *Bathyarchaeum*.

### Description of the class *Bathyarchaeia*

Ba.thy.ar.chae'ia (N.L. fem. n. *Bathyarchaeum*, type genus of the class; N.L. neut. n. suff. *-ia*, ending to denote a class; N.L.neut. pl. n. *Bathyarchaeia*, class of the genus *Bathyarchaeum*).

GTDB 207 supports delineation of the class *Bathyarchaeia* (f__BA1) with RED being equal to 0.514. The nomenclatural type of the class is the genus *Bathyarchaeum*.

## Data availability statement

The datasets presented in this study can be found in online repositories. The names of the repository/repositories and accession number(s) can be found in the article/Supplementary material.

## Author contributions

MK, AM, DM, and AK: investigation. MK, AM, and AS: writing—original draft preparation. AS and MK: writing—reviewing and editing. MK and AM: visualization. AM: funding acquisition. All authors contributed to the article and approved the submitted version.
