# Supplementary material for: Phenotypic and genomic characterization of Bathyarchaeum tardum gen. nov., sp. nov., a cultivated representative of the archaeal class Bathyarchaeia
Source: Front Microbiol. 2023 Aug 22;14:1214631. doi: 10.3389/fmicb.2023.1214631 (PMC10477458; doi:10.3389/fmicb.2023.1214631)
Supplement: Supplementary file 1 [file Data_Sheet_1.pdf]

Phenotypic and genomic characterization of *Bathyarchaeum tardum* gen. nov., sp. nov., a cultivated representative of the archaeal class *Bathyarchaeia*

Maria A. Khomyakova[1*], Alexander Y. Merkel[1], Dana D. Mamiy[2], Alexandra A. Klyukina[1], Alexander I. Slobodkin[1]

*** Correspondence:** mary_klimova@mail.ru

SUPPLEMENTARY MATERIALS

Supplementary Text 1.
The process of *Bathyarchaeia* isolation

The enrichment culture M17C was set up by inoculation of the sample of anaerobic sediment into anaerobic medium containing MAC (10 mM of 2-methoxybenzoic acid, 2-methoxyphenol or 3,4-dimethoxybenzoic acid (DMB)) and 0.5 g l^{-1} ampicillin to suppress bacterial growth. Sample without the addition of MAC, cultivated on basal medium with yeast extract (0.1 g l^{-1}) and ampicillin (0.5 g l^{-1}), was used as control. In the control without any substrate, growth was poor, and yeast extract was added to the control instead of MACs as the substrate equivalent.

For M17C, where the natural sediment contained only 0.4% of *Bathyarchaeia* (1.76E+05 of 16S rRNA gene copies per ml), the enrichment culture cultivated on 10 mM DMB demonstrated gradually the growth of small cocci, subcultured within each 2 months. After the first transfer the amount of 16S rRNA gene copies per ml of enrichment culture was the highest and composed 2.30E+07 compared with control (9.62E+04), and gradually decreased by the third transfer to 5.00E+06. However, the relative abundance of *Bathyarchaeia* after 3 transfers reached 74% in term of the all microbial composition, especially of bathyarchaeal genome subgroup-8 (Supplementary Figure S1). Bacterial composition was rich in *Dethiobacter* uncultured group (8%), *Izhimaplasmataceae* and *Acholeplasma* representatives of *Tenericutes* (9% integrally) (Biosample Accession number in Genbak is SAMN34140870). By the third transfer, the number of 16S rRNA gene copies per ml of bathyarchaeal enrichment culture reached 5.0E+06 and was approximately 3.5 times higher than the control without DMB (1.49E+06 of 16S rRNA gene copies per ml). The bacterial component in both test and control were represented mainly by the species of phylum *Tenericutes* (uncultivated *Izhimaplasmataceae* and *Mariniplasma* sp), which subsequently suppressed the growth of the *Batyarchaeia* group. Since M17C demonstrated a good growth in this probe, the first metagenome of this sample was sequenced (M17C, SAMN30120881), and further attempts to

isolate a pure culture of *Bathyarchaeia* were proceeded. However, after the third transfer, the application of the identified techniques did not help in the subsequent cultivation of the enrichment culture: no obvious growth was observed. The isolation of uncultured organoheterotrophic *Mariniplasma* sp. (Watanabe et al. validated its closest relative *Mariniplasma anaerobium* gen. nov. sp. nov. in 2021) let us determine the sensitivity of interfering group to antibiotics (streptomycin and lincomycin) and utilized protein substrates (casamino acids did not support the growth of *Mariniplasma* sp). Subsequent cultivation of *Bathyarchaeia* enrichment culture with a mixture of streptomycin and lincomycin (0.05 g l^{-1} each) and the replacement of the yeast extract with casamino acids (0.1 g l^{-1}) together with the addition of sterile diluted natural sample from Golubitskoye lake (0.2 ml 10 ml $^{-1}$ (v/v)) made it possible to obtain finally a highly purified culture of *Bathyarchaeia*, where the relative abundance of this uncultivated group reached 96–98% of the entire microbial community (Supplementary Figure S1). Attempts to cultivate this culture on solid medium with agar or gelrite as described in Hu et al., 2021, were unsuccessful.

Currently, the growth rate of initial culture on mixture of DMB and yeast extract takes 5-6 months from one transfer to the next; the use of only one of these substrates leads to inhibition of culture growth. However, during a 5-year purification of this enrichment culture ten subsequent transfers with two serial ten-fold dilutions with evident microbial growth in the highest positive dilution (10^{-7}) have been performed (see First cultivation Line in Supplementary Figure S1). Through subsequent transfers, we were able to eliminate bacterial population to 0.4–0.8%, enabling us to obtain nearly co-culture of the target archaeon $M17C^{Ts}$ and subsequently isolated *Methanocalculus alkaliphilus*, which could reach 1–12% depending on the cultivation variant. Moreover, valid transmission electron microscopy was applied at this stage of *Bathyarchaeia* isolation. In another cultivation line without serial dilutions the growth of enrichment culture is better, *Bathyarchaeia* isolate reaches 97.8% of enrichment culture (such a high yield was supported by further metagenomic analysis (metagenome M17C-73, SAMN33973097), however, there is a much greater variety of methanogenic archaea (see Second cultivation Line in Supplementary Figure S1). FISH was also applied at this stage of *Bathyarchaeia* isolation.

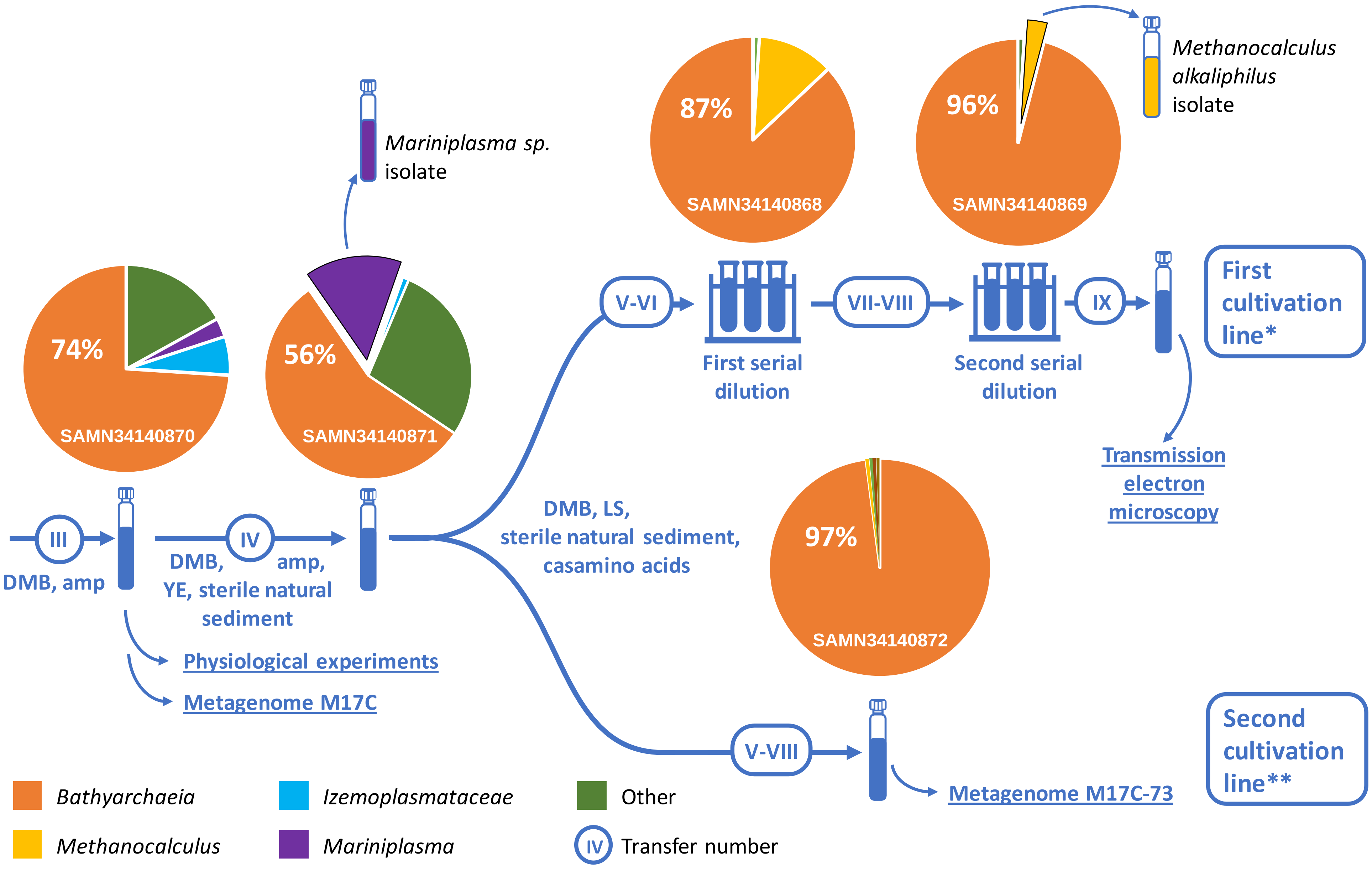
Mariniplasma sp. isolate
87%
SAMN34140868
96%
SAMN34140869
Methanocalculus alkaliphilus isolate
74%
SAMN34140870
56%
SAMN34140871
V-VI
First serial dilution
VII-VIII
Second serial dilution
IX
First cultivation line*
Transmission electron microscopy
DMB, LS, sterile natural sediment, casamino acids
97%
SAMN34140872
III
DMB, amp
IV
DMB, amp, YE, sterile natural sediment
Physiological experiments
Metagenome M17C
V-VIII
Metagenome M17C-73
Second cultivation line**
Bathyarchaeia
Izemoplasmataceae
Other
Methanocalculus
Mariniplasma
IV Transfer number

Supplementary Figure S1. Schematic diagram of the purification process for strain M17C^{Ts} starting from the third transfer: III-IX amount of transfers; *Mariniplasma* sp. and *Methanocalculus alkaliphilus* were isolated after fourth and eight transfer correspondingly; amp ampicillin (0.5 g l^{-1}), LS lincomycin and streptomycin (0.05 g l^{-1} each); YE yeast extract (0.1 g l^{-1}); DMB 3,4-dimethoxybenzoic acid (10mM); sterile natural sediment was diluted 1:10 with cultural medium and added 0.2 ml 10 ml^{-1} of culture; both serial ten-fold dilutions were grown up to the highest positive dilution

(10^{-7}), profiling for the V4 hypervariable region of the 16S rRNA gene was made from the 6th positive dilution.

*First cultivation line is extremely slowly-grown and represented predominantly *by Bathyacrhaeia* isolate and *Methanocalculus alkaliphilus*;

**Second cultivation line is better and faster growing, represented by *Bathyarchaeia* isolate and different methanogenic groups, including uncultured.

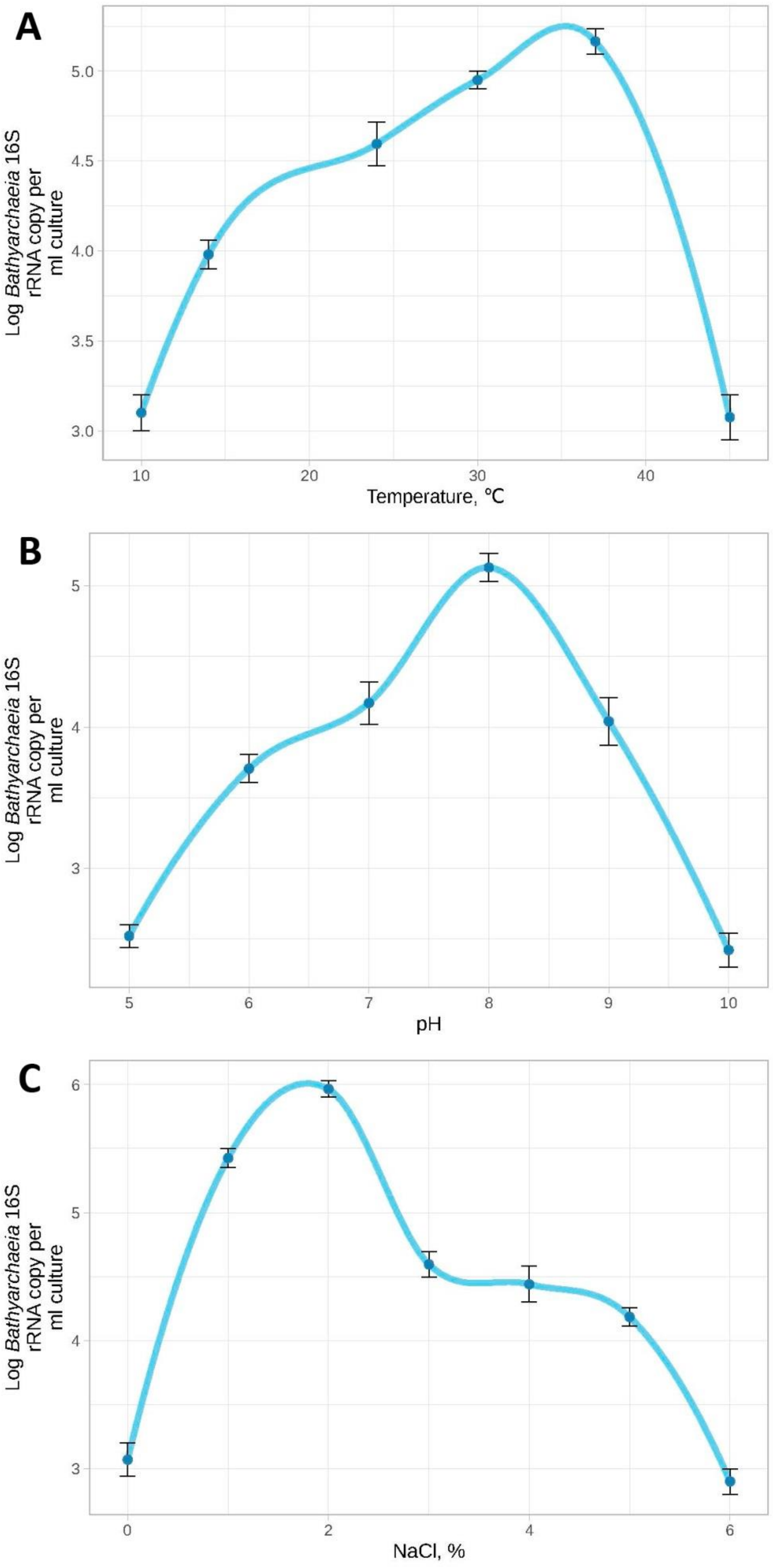

Supplementary Figure S2 The effect of physicochemical parameters on the growth of strain M17C^{Ts}: *(A)* incubation temperature; *(B)* pH; *(C)* NaCl concentration.

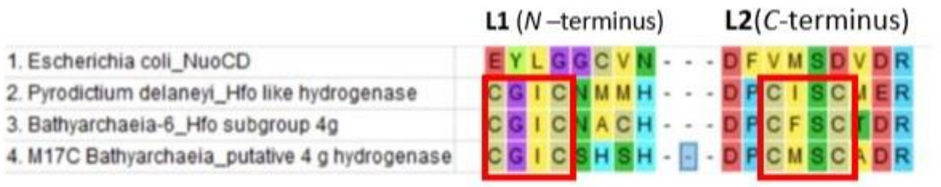

Supplementary Figure S3. Comparison of the [NiFe]-binding motifs (L1 and L2) in the large subunit of putative M17C^{Ts} hydrogenase subgroup 4g with the corresponding amino acid residues of its known homologs of the subgroup 4g and of Nuo complex (*E. coli*) (Vignais and Billoud, 2007). The four cysteine residues that coordinate the [NiFe] cluster are framed in red. Gene numbers taken for alignment, are WGM88738.1 (Genbank, M17C), P33599.3 (UniProtKB/Swiss-Prot, *E. coli*), 2574365692 (IMG, *Bathyarchaeia* of subgroup 6), 2775380982 (IMG, *Pyrodictium delaneyi*)

Supplementary Table S1. Main assembly quality indicators for M17C and M17C-73 metagenomes.

Assembly quality indicators	M17C	M17C-73-01
# contigs	1	12
Largest contig	2152572	521597
Total length	2139529	2152572
GC (%)	38.14	38.14
L50	1	3
N50	2152572	310348
Completeness	98.28	97.82
Contamination	0.934	0.934
complete rRNAs (5S, 16S, 23S)	1, 1, 1	1, 1, 1
tRNAs	46	46
GenBank accession	CP122380	GCA_029762755.1

Supplementary Table S2. Annotation of genes for the strain M17C (GenBank ID JANKMT000000000)

Predicted protein	Gene name	Genbank accession number	KEGG
Degradation of methoxylated and hydroxylated aromatic compounds			
[methyl-Co(III) methoxylated-aromatic-compound-specific corrinoid protein]---tetrahydromethanopterin methyltransferase (MTII) of *O*-demethylase	mtoA, mtvA, mtrH	WGM89984.1	K25917, K25915, K00584
Putative corrinoid protein Co-methyltransferase (MTI) of *O*-demethylase	mtoB, mtvB	WGM89986.1	K25916
Corrinoid protein of *O*-demethylase	mtoC, mtvC	WGM89983.1	
Activating enzyme of *O*-demethylase	mtoD	ND	
benzoyl-CoA reductase subunit BcrA	bcrA, badF	WGM90204.1	K04114
benzoyl-CoA reductase subunit BcrB	bcrB, badE	ND	K04113
Putative benzoyl-CoA reductase subunit BcrC	bcrC, badD	WGM90206.1	K04112
Putative benzoyl-CoA reductase subunit BcrD	bcrD, badG	WGM90205.1	K04115
4-hydroxybenzoate--CoA/benzoate--CoA ligase	hbaA, hcrL	ND	
3-hydroxybenzoyl-CoA ligase	hbcL	ND	K20458
4-hydroxybenzoyl-CoA reductase subunit A	hcrA	ND	K04108
4-hydroxybenzoyl-CoA reductase subunit B	hcrB	ND	K04109
4-hydroxybenzoyl-CoA reductase subunit C	hcrC	ND	K04107
Wood-Ljungdahl pathway			
anaerobic carbon-monoxide dehydrogenase, CODH/ACS complex subunit alpha	cdhA	WGM90611.1	K00192
acetyl-CoA decarbonylase/synthase, CODH/ACS complex subunit beta	cdhC	WGM90654.1	K00193
acetyl-CoA decarbonylase/synthase, CODH/ACS complex subunit gamma	cdhE, acsC	WGM90614.1	K00197
anaerobic carbon-monoxide dehydrogenase, CODH/ACS complex subunit epsilon	cdhB	WGM90612.1	K00195
acetyl-CoA decarbonylase/synthase, CODH/ACS complex subunit delta	cdhD, acsD	WGM90613.1	K00194
formylmethanofuran dehydrogenase subunit A	fwdA	WGM88596.1	K00200
Putative formylmethanofuran dehydrogenase subunit B	fwdB	WGM89749.1	K00201
formylmethanofuran dehydrogenase subunit C	fwdC	WGM88595.1	K00202
Putative formylmethanofuran dehydrogenase subunit D	fwdD	WGM90662.1	K00203

formylmethanofuran dehydrogenase subunit E	fwdE	ND	K11261
formylmethanofuran dehydrogenase subunit	fwdF	WGM89817.1	K00205
formylmethanofuran--tetrahydromethanopterin N-formyltransferase	ftr	WGM88604.1, WGM89568.1	K00672
methenyltetrahydromethanopterin cyclohydrolase	mch	WGM88600.1	K01499
methylenetetrahydromethanopterin dehydrogenase	mtd	WGM88599.1	K00319
5,10-methylenetetrahydromethanopterin reductase	mer	WGM89521.1	K00320
acetyl-CoA synthetase	acs	WGM89748.1	K01895
tetrahydromethanopterin S-methyltransferase subunit H	mtrH	WGM89984.1	K00584
Formate dehydrogenase	fdh	ND	K00122
Glycolysis/Gluconeogenesis			
glucokinase	glk	WGM90304.1	K25026
glucose/mannose-6-phosphate isomerase	pgi-pmi	WGM88699.1	K15916
6-phosphofructokinase 1	pfka	WGM90127.1	K00850
fructose-bisphosphate aldolase	fbpa	WGM89761.1	K01624
triosephosphate isomerase	tpi	WGM88714.1	K01803
glyceraldehyde 3-phosphate dehydrogenase	gapdh	WGM90221.1	K00134
phosphoglycerate kinase	pgk	WGM90222.1	K00927
2,3-bisphosphoglycerate-independent phosphoglycerate mutase	apgm	WGM88986.1, WGM88905.1	K15635
enolase	eno	WGM89587.1	K01689
pyruvate kinase	pyk	WGM89484.1	K00873
phosphoenolpyruvate carboxykinase	pck	WGM89114.1	K01596
fructose-1,6-bisphosphatase II	glpX	WGM89182.1	K02446
Glucose-6-phosphatase	g6pc	ND	K01084
Pyruvate ferredoxin oxidoreductase subunit A	porA	WGM89687.1	K00169
Putative pyruvate ferredoxin oxidoreductase subunit B	porB	WGM89686.1	K00170
Putative pyruvate ferredoxin oxidoreductase subunit D	porD	WGM89688.1	K00171
Pyruvate ferredoxin oxidoreductase subunit G	porG	WGM89689.1	K00172
Protein degradation			
Branched-chain amino acid aminotransferase	ilvE	WGM89361.1	K00826
Acetolactate synthase I/II/III large subunit	ilvB, ilvG, ilvI	WGM89064.1	K01652
Histidinol-phosphate/aromatic aminotransferase	hisC	WGM89080.1	K00817
Imidazole glycerol-phosphate synthase subunit HisH, glutamine amidotransferase	hisH	WGM89077.1	K02501

Putative 4-aminobutyrate aminotransferase	puuE	WGM89691.1	K00823
2-oxoglutarate/2-oxoacid ferredoxin oxidoreductase subunit alpha	korA, oorA, oforA	WGM88769.1	K00174
Putative 2-oxoglutarate/2-oxoacid ferredoxin oxidoreductase subunit beta	korB, oorB, oforB	WGM88770.1	K00175
Putative 2-oxoglutarate ferredoxin oxidoreductase subunit delta	korD, oorD	WGM89817.1	K00176
2-oxoglutarate ferredoxin oxidoreductase subunit gamma	korC, oorC	ND	K00177
aldehyde ferredoxin oxidoreductase	aor	WGM89453.1	K03738
Energetic metabolism			
V-type ATP synthase subunit A	ATPVA, ntpA, atpA	WGM89702.1	K02117
V-type ATP synthase subunit B	ATPVB, ntpB, atpB	WGM89703.1	K02118
V-type ATP synthase subunit C	ATPVC, ntpC, atpC	ND	K02119
V-type ATP synthase subunit D	ATPVD, ntpD, atpD	WGM89704.1	K02120
V-type ATP synthase subunit E	ATPVE, ntpE, atpE	WGM89710.1	K02121
V-type ATP synthase subunit F	ATPVF, ntpF, atpF	ND	K02122
V-type ATP synthase subunit I	ATPVI, ntpI, atpI	WGM89708.1	K02123
V-type ATP synthase subunit K	ATPVK, ntpK, atpK	WGM89707.1	K02124
Respiratory complex I:			
NADH-quinone oxidoreductase subunit A	nuoA	WGM88890.1	K00330
NADH-quinone oxidoreductase subunit B	nuoB	WGM88889.1 WGM88739.1	K00331
NADH-quinone oxidoreductase subunit C	nuoC	WGM88888.1 WGM88731.1	K00332
Putative NADH-quinone oxidoreductase subunit D	nuoD	WGM88738.1	K00333
Putative NADH-quinone oxidoreductase subunit E	nuoE	WGM89840.1	K00334
Putative NADH-quinone oxidoreductase subunit F	nuoF	WGM89841.1	K00335
Putative NADH-quinone oxidoreductase subunit G	nuoG	WGM89842.1	K00336
NADH-quinone oxidoreductase subunit H	nuoH	WGM88887.1 WGM88737.1	K00337
NADH-quinone oxidoreductase subunit I	nuoI	WGM88886.1	K00338
NADH-quinone oxidoreductase subunit J	nuoJ	ND	K00339
NADH-quinone oxidoreductase subunit K	nuoK	WGM88884.1	K00340
NADH-quinone oxidoreductase subunit	nuoL	WGM88883.1	K00341

L			
NADH-quinone oxidoreductase subunit M	nuoM	WGM88882.1	K00342
NADH-quinone oxidoreductase subunit N	nuoN	WGM88881.1	K00343
Ethanol fermentation			
Alcohol dehydrogenase, class IV	adh	ND	K00001
Alcohol dehydrogenase, propanol-preferring	adhP	ND	K13953
Alcohol dehydrogenase	yiaY	ND	K13954
aldehyde ferredoxin oxidoreductase	aor	WGM89453.1	K03738
Cobalamine B12 biosynthesis (anaerobic pathway)			
sirohydrochlorin cobaltochelatase	cbik	ND	K02190
sirohydrochlorin cobaltochelatase	cbiX	WGM88847.1	K03795
sirohydrochlorin cobalto/nickelchelatase	cfbA	ND	K22011
precorrin-6A/cobalt-precorrin-6A reductase	cobK-cbiJ	ND	K05895
Putative precorrin-8X/cobalt-precorrin-8 methylmutase	cobH-cbiC	WGM88847.1	K06042
cobalt-precorrin-5B (C1)-methyltransferase	cbiD	WGM88848.1	K02188
cobalt-precorrin 5A hydrolase	cbiG	WGM88853.1	K02189
cobalt-precorrin-6B (C15)-methyltransferase	cbiT	WGM88850.1	K02191
cobyrinic acid a,c-diamide synthase	cobI-cbiA	WGM89997.1	K02224
precorrin-2/cobalt-factor-2 C20-methyltransferase	cobI-cbiA	WGM88851.1	K03394
cobalt-precorrin-7 (C5)-methyltransferase	cbiE	WGM88849.1	K03399
precorrin-3B C17-methyltransferase / cobalt-factor III methyltransferase	cobG, cbiH	WGM90670.1	K05934
precorrin-4/cobalt-precorrin-4 C11-methyltransferase	cobM, cbiF	WGM88852.1	K05936
uroporphyrinogen III methyltransferase / synthase	cobA-hemD	WGM88841.1	K13542
precorrin-2 dehydrogenase	sirC	WGM88845.1	K24866
adenosylcobinamide-phosphate synthase	cbiB-cobD	WGM88869.1, WGM90002.1	K02227
adenosylcobinamide-GDP ribazoletransferase	cobS, cobV	WGM90637.1, WGM89982.1	K02233
cob(I)alamin adenosyltransferase	mmab	ND	K00798
cob(I)alamin adenosyltransferase	cobA, btuR	ND	K19221
adenosylcobyric acid synthase	cobQ, cbiP	ND	K02232
cobalamin biosynthesis protein CobC	cobC	ND	K02225
adenosylcobinamide kinase / adenosylcobinamide-phosphate guanylyltransferase	cobP, cobU	ND	K02231
nicotinate-nucleotide--dimethylbenzimidazole phosphoribosyltransferase	cobU, cobT	ND	K00768

alpha-ribazole phosphatase	cobC, phpB	ND	K02226
ribonuclease H / adenosylcobalamin/alpha-ribazole phosphatase	rhnA-cobC	ND	K22316
CoF420 biosynthesis			
coenzyme F420-0:L-glutamate ligase / coenzyme F420-1:gamma-L-glutamate ligase	cofE	WGM88566.1	K12234
5-amino-6-(D-ribitylamino)uracil---L-tyrosine 4-hydroxyphenyl transferase	cofH	ND	K11781
7,8-didemethyl-8-hydroxy-5-deazariboflavin synthase	cofG	ND	K11780
2-phospho-L-lactate/phosphoenolpyruvate guanylyltransferase	cofC	ND	K14941
LPPG:FO 2-phospho-L-lactate transferase	cofD	ND	K11212
Biosynthesis of guanine ribonucleotides GDP/GTP			
GMP synthase (glutamine-hydrolysing)	guaA	WGM89764.1, WGM89329.1	K01951
Inosine-5'-monophosphate dehydrogenase	guaB	ND	K00088
guanylate kinase	gmk	ND	K00942
nucleoside-diphosphate kinase	ndk	ND	K00940
adenylate/nucleoside-diphosphate kinase	ak9	ND	K18533
Biosynthesis of pyrimidine deoxyribonucleotides dTTP			
nucleoside-diphosphate kinase	ndk	ND	K00940
adenylate/nucleoside-diphosphate kinase	ak9	ND	K18533
Deoxyuridine 5'-triphosphate nucleotidohydrolase	dut	ND	K01520
ribonucleoside-diphosphate reductase alpha chain	nrdA	WGM89089.1	K00525
thymidylate synthase	thyA	WGM90605.1	K00560
dTMP kinase	tmk	WGM88809.1	K00943

References:

Hu, H., Natarajan, V. P., and Wang, F. (2021). Towards enriching and isolation of uncultivated archaea from marine sediments using a refined combination of conventional microbial cultivation methods. Mar. Life Sci. Technol. 3, 231–242. doi: 10.1007/s42995-021-00092-0

Vignais, P. M., and Billoud, B. (2007). Occurrence, classification, and biological function of hydrogenases: an overview. Chem. Rev. 107, 4206–4272. doi: 10.1021/cr050196r

Watanabe, M., Kojima, H., Okano, K., and Fukui, M. (2021). *Mariniplasma anaerobium* gen. nov., sp. nov., a novel anaerobic marine mollicute, and proposal of three novel genera to reclassify members of *Acholeplasma* clusters II-IV. Int. J. Syst. Evol. Microbiol. 71. doi: 10.1099/ijsem.0.005138
